# Linear and Angular Distyrylpyrazines with Terminal Donor Groups: Synthesis, Solvatochromism, and Acidochromism of the Electronic Spectra

**DOI:** 10.5402/2011/589012

**Published:** 2011-04-20

**Authors:** Volker Schmitt, Janina Fischer, Heiner Detert

**Affiliations:** Institute for Organic Chemistry, Johannes Gutenberg-University Mainz, Duesbergweg 10–14, 55099 Mainz, Germany

## Abstract

A series of linear and angular distyrylpyrazines and lateral donor groups has been prepared by aldol condensation between dimethylpyrazines and the appropriate aromatic aldehyde. The optical absorption and emission properties of these systems were studied in different solvents and media. The materials display a strong solvatochromism of the emission that is reflected by large red shifts of their fluorescence emission maxima on increasing the solvent polarity. This behaviour suggests a highly polar emitting state, which is characteristic of compounds that undergo an internal charge transfer upon excitation. Upon protonation, the UV-vis spectra are altered, and the fluorescence intensity of the neutral compound vanishes. These molecules can be used as colorimetric and luminescence polarity and pH sensors.

## 1. Introduction

*π*-Conjugated oligomers [1] like oligo(phenylenevinylene)s [2, 3] and their heterocyclic derivatives [4–7] have attracted much interest for the application as active materials in organic light-emitting devices. The substitution of conjugated polymers [8] and oligomers with side chains of different types has been widely used to improve solubility and mechanical behaviour of these materials. Another important aspect is the directed tuning of their electrical and optical properties for applications in optoelectronic devices [9, 10]. The combination of electron-pair-donating (EPD) and electron-pair-accepting (EPA) substituents can lead to materials for nonlinear optical applications such as second harmonic generation or two-photon absorption [11, 12]. In this context, alkoxy and amino groups, being efficient and typical donor groups, are often combined with strong acceptors like nitriles. In addition to nitriles, fluorination, and other electron-withdrawing groups on a benzene ring, electron-deficient heterocycles like oxadiazole, triazine, or pyridazine are also suitable as acceptors in *π*-conjugated oligomers. The interaction of these compounds with the environment can result in significant spectroscopic changes, useful for sensing purposes [13, 14]. A few alkoxy-substituted distyrylpyrazines had been prepared [15–17] and investigated for optoelectronic devices, as emitting materials for lasing purposes [18, 19], and also as two-photon absorbing dyes [20]. The generally applied synthetic routes to styrylpyrazines are based on the Lewis acid- catalyzed condensation of aromatic aldehydes [21] and on the deprotonation of methylpyrazines and aldol condensation with aromatic aldehydes. Several variations like Siegrist reaction [22] or phase-transfer conditions [23] proved to be advantageous in specific preparations. 

The electron-donating ability of amines is inverted upon protonation or quaternization, particularly the pyridinium ion is a common acceptor unit [24, 25], but ammonium ions have also received considerable attention [26]. The influence of environmental conditions on the electron donating or accepting capability of amino groups and azines can be used to alter the optical properties of a chromophore by tuning the strength of the nitrogen-containing donor (amines) and acceptor (azines) groups. Complexation with Lewis acids or protonation has been shown to cause significant changes of the UV/Vis absorption and emission spectra of *π*-conjugated chromophores with basic sites on the terminal positions [27–29]. As triarylamines are very poor bases, they are protonated only under extremely forcing conditions. Therefore, donor groups based on triphenylamine or phenylcarbazole will preserve their electron-donating effect even in the presence of strong acids. Quadrupolar fluorophores with a pronounced donor-acceptor-donor substitution are interesting candidates for sensing applications for polarity, amines, ions, and protons [30, 31]. Furthermore, dyes with this electronic structure and a solvatochromism of the fluorescence are good candidates for highly efficient two-photon absorption in the NIR [11, 12, 20].

As part of our interest in fluorophores with switchable optical properties and efficient two-photon absorption [12, 32, 33], we report here the synthesis of linear 2,5-distyrylpyrazines (DSP) with lateral donor groups. The pyrazine acts as an electron acceptor, resulting in a donor-acceptor-donor electronic structure of the chromophores. For comparison purposes, some V-shaped isomers are included. Since the donors are methoxy or carbazole units, protonation can occur only at the pyrazine as central ring of these quadrupolar and dipolar chromophores. The impact of the environment on the electronic spectra is also reported; solvent polarity and protonation have a distinct effect on the absorption or emission behaviour.

## 2. Synthesis

Linear distyrylpyrazines **1**–**7** were prepared via twofold condensation of aromatic aldehydes **12**–**18** with 2,5-dimethylpyrazine (**11**) or 2,5-dimethylpyrazine-*N*,*N*-dioxide (**19**).

 The methyl groups in **11** are highly activated; therefore, potassium-*t*-butylate in DMF is suitable to deprotonate the methyl groups and to induce a direct aldol condensation on both sides of **11** with donor-substituted benzaldehydes **12**–**17** to form **1**, **3–7**. 

**Scheme 2:**
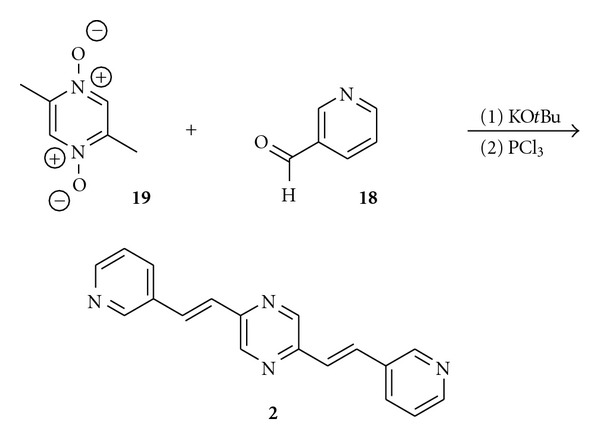
Synthesis of Bis(pyridylethenyl)pyrazine 2.

An activation of the pyrazine **11** via oxidation to the bis-*N*-oxide **19** was necessary for the condensation with pyridyl carbaldehyde **18** followed by reduction of the bis-*N*-oxide with PCl_3_ to yield bis(*β*-pyridylethenyl)pyrazine **2 **(64%) (Scheme 2). 

**Scheme 3:**
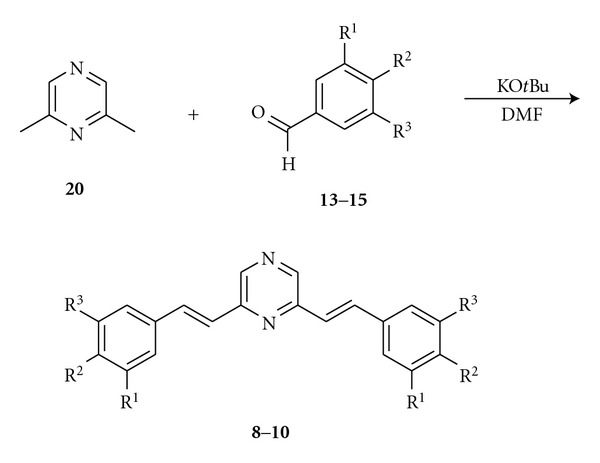
Synthesis of angular distyrylpyrazines **8–10**.

The V-shaped chromophores **8**–**10** were prepared similarly by condensation of 2,6-dimethylpyrazine (**20**) with methoxybenzaldehydes **13**–**15 **(Scheme 3). These condensations are a very fast access to distyrylpyrazines, nevertheless, the yields are at maximum moderate (4–55%, 64% for **2**). Chromatography on silica gel followed by recrystallization from chloroform/methanol gave pure **1**–**10** (Table 1) without detectable traces of *cis*-isomers. 

## 3. Electronic Spectra

The solid distyrylpyrazines (DSPs) **1–10** are yellow to orange crystalline compounds with a low to moderate solubility in common solvents like toluene or chloroform. The colorless **(2)** or yellow solutions **(1, 3–10)** are highly fluorescent. UV-vis absorption spectra were obtained from 10^−5^ M solutions in cyclohexane, toluene, dichloromethane, acetonitrile, and ethanol and from solutions in dichloromethane containing 10^−5^ M–1 M trifluoroacetic acid (TFA). Solutions with a concentration of 10^−7^ M **1–10** were used for the measurement of the fluorescence spectra. Excitation wavelength was *λ*_exc_ = 345 nm. Spectroscopic data are collected in Tables 2 and 3.

The UV/Vis-absorption spectra of these distyrylpyrazines in cyclohexane are dominated by a low-energy band with a maximum around *λ* = 383 nm (Table 2). The main absorption band of **10** peaks at *λ* = 316 nm, but a second maximum at *λ* = 376 nm and an intense shoulder at *λ* = 400 nm are visible. Lateral alkoxy or carbazolyl substitution on the distyrylpyrazine system results in an electronic donor-acceptor-donor structure and shifts the excitation to lower energies. Within the series **1–6** (in dichloromethane), the bathochromic shift is about 31 nm (Δ*ν* = 2025 cm^−1^). The red shift of the absorption of **7** (*λ*_max⁡_ = 427 nm, DCM) relative to its isomer **6** (*λ*_max⁡_ = 405 nm, DCM) results from the extension of the conjugated system by indolo annulation. Compared to the fundamental chromophore **1**, the auxochromic effect of the donor substitution also causes a strong hyperchromism, for example, **1**: *ε*_max⁡_ = 43537 L/mol*cm, **6** : *ε*_max⁡_ = 61976 L/mol*cm.

A comparison in the methoxy series shows the expected bathochromic shift of the excitation by increasing the donor strength of the lateral rings from a 4-methoxy **(3)** to a 3,4-dimethoxy substitution **(4)**, but a hypsochromic shift results when the third methoxy group **(5)** is attached to the *π*-system. An even more pronounced hypsochromism with increasing donor substitution occurs in the angular series. The absorption maximum of the di(*p*-methoxystyryl)pyrazine **8** peaks at *λ*_max⁡_ = 400 nm (DCM); further donor groups shift the maximum to *λ*_max⁡_ = 395 nm **(9)** and 381 nm **(10)**. Semiempirical calculations on **8** and **9** also give a small hypsochromic shift of Δ*λ* = 6 nm with the additional *meta*-methoxy groups. It should be noted that the main excitation band of the linear chromophores compared to their angular isomers is only slightly shifted to lower energies. As the excitation of related distyrylbenzene systems with a quadrupolar electronic structure can be localized on one part of the chromophore [34], a formal elongation of the *π*-system has only minor effect. In our case, the excitation can be regarded as localized on a methoxystyrylpyrazine, and the second styryl group provokes relatively small variations of the orbital energies of this fragment.

### 3.1. Fluorescence Spectra

All compounds are strongly fluorescent in dilute solution (Table 2). Donor substitution results in bathochromic shifts, but the range of these shifts is much larger compared to the shifts of the excitation spectra. The pyridyl derivative **2** emits with *λ*_max⁡_^F^ = 423 nm (DCM), the phenyl analogue **1** with *λ*_max⁡_^F^   = 432 nm, and six additional methoxy donors (**5**: *λ*_max⁡_^F^ = 489 nm) or two 9-carbazolyl groups (**6**: *λ*_max⁡_^F^ = 495 nm) shift the emission maximum from the violet to the blue-green. The linear and angular dyes with a *p*-methoxy group are very similar in their emission (**3**: *λ*_max⁡_^F^ = 465 nm; **8**: *λ*_max⁡_^F^ = 463 nm), and the influence of further auxochromic groups on the emission of the angular isomers is rather small (**10**: *λ*_max⁡_^F^ = 474 nm).

### 3.2. Solvatochromism

**Figure 1:**
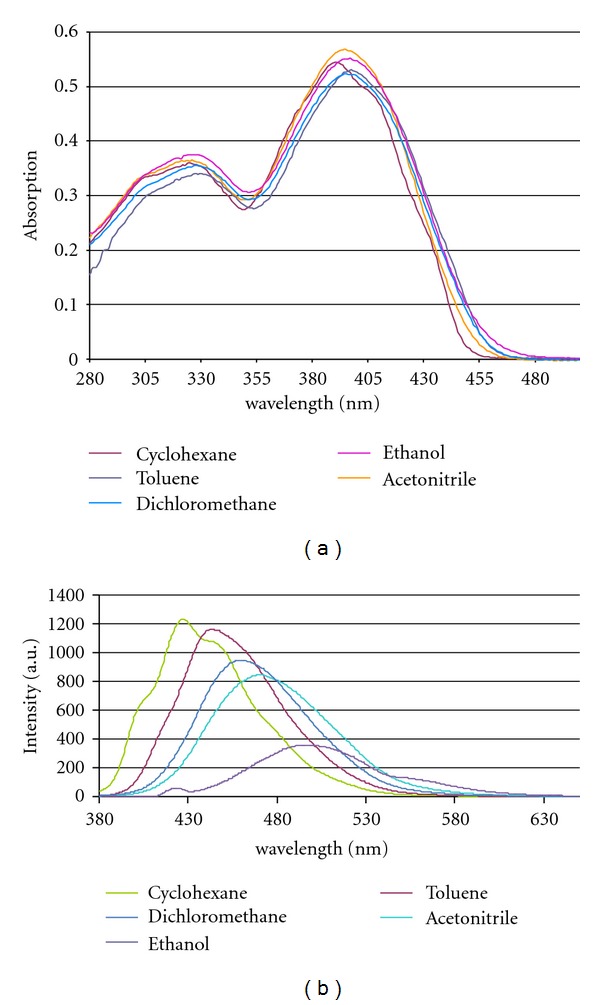
Solvent-depending UV-vis absorption and fluorescence spectra of angular bis(3,4-dimethoxystyryl)pyrazine **9.**

Only minor shifts of the absorption of these dyes result from changing the solvent polarity. Within the solvents used, variations of *λ*_max⁡_ are in the range of 6 nm, only **6** is stronger influenced (Δ*λ* ≤ 10 nm). This was expected for the linear, centrosymmetric D-A-D chromophores, but surprisingly, the influence of the solvent on the absorption of the noncentrosymmetrical angular derivatives **8–10** is comparably small. The calculated dipole moments of angular DSPs increase in the series **8** (0.122 D), **10** (1.79 D), and **9** (2.19 D). The similarity of the solvatochromism of linear and V-shaped DSPs is in accordance with a localization of the excitation preferentially on a donor-styrylpyrazin segment of the *π*-system [34]. A typical series of electronic spectra in various solvents is depicted in Figure 1.

Whereas the absorption of these dyes is nearly unbiased by solvent polarity, a strong positive solvatochromism was observed in the fluorescence spectra of the linear, quadrupolar chromophores. Within the solvent range cyclohexane–ethanol, the bathochromic shift of distyrylpyrazines without donor substituents **(1, 2)** is below Δ*λ* = 20 nm (Δ*ν* = 1011 cm^−1^) with the largest shift in the protic solvent ethanol. But donor substitution results in an intensified sensitivity of the fluorophore towards solvent polarity. A terminal *p*-methoxy substitution **(3)** extends the shift to Δ*λ* = 43 nm (Δ*ν* = 2032 cm^−1^), a 3,4-dimethoxy substitution (**4**) to Δ*λ* = 53 nm (Δ*ν* = 2361 cm^−1^) and to Δ*λ* = 65 nm (Δ*ν* = 2816 cm^−1^) for the 3,4,5-trimethoxy derivative **5**. The largest displacement shows the carbazole-terminated chromophore **6** with Δ*λ* = 69 nm (Δ*ν* = 2942 cm^−1^). Contrary to the weak solvatochromism of the absorption of the angular, dipolar distyrylpyrazines, their emission is highly sensitive towards polarity changes; the solvatochromism of **8** is nearly identical to that of its linear analogue **3**, but further donor substitution results in increased solvatochromic displacements of the emission of Δ*λ* = 62 nm (Δ*ν* = 3339 cm^−1^) for **9** (Figure 1) and of Δ*λ* = 89 nm (Δ*ν* = 4092 cm^−1^) for **10**. As observed for other linear and angular fluorophores [7], the positive solvatochromism is accompanied by reduced fluorescence intensities of the linear dyes and more pronounced for their V-shaped isomers. Comparing solutions of **8–10** in cyclohexane and ethanol, the fluorescence intensities drop to ca. 50% for **8**, 25% for **9,** and less than 10% for **10**.

### 3.3. Acidochromism

**Figure 2:**
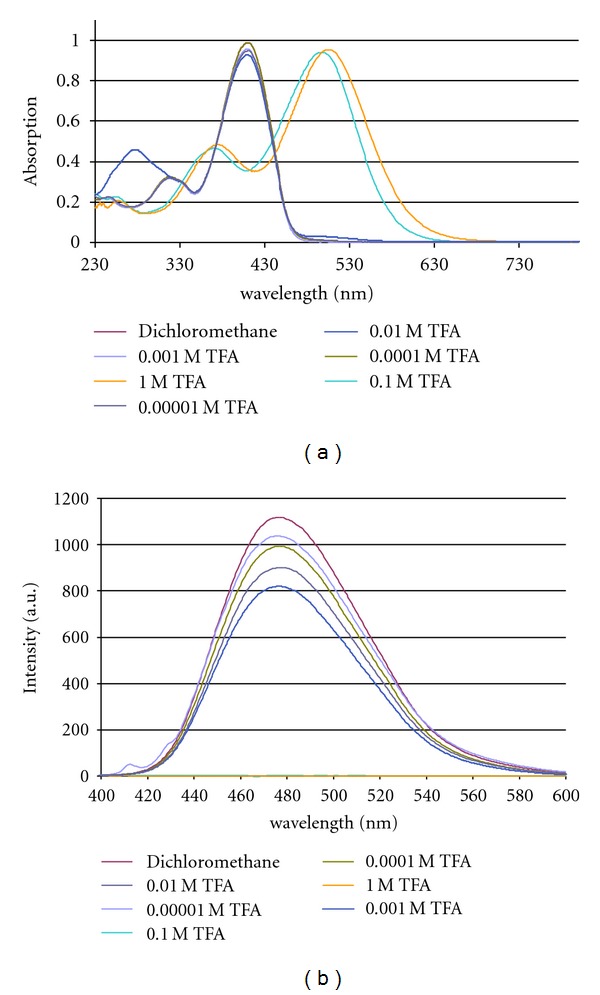
Acidochromism of absorption and fluorescence of linear bis(3,4- dimethoxystyryl)pyrazine **4** in the presence of TFA.

The distyrylpyrazine chromophore is an intrinsically basic compound. As the pyrazine is only a very weak base (pK_B_ = 13.4) [36], protonation of **1** starts only with 10^−2^ M TFA in dichloromethane. This protonation strongly enhances the electron acceptor capability of the heterocycle, and the long-wavelength absorption band of **1** is gradually displaced from *λ*_max⁡_ = 383 nm to *λ*_max⁡_ = 450 nm (Table 3). Donor substitution results in an even stronger redshift of the absorption upon protonation. For these compounds **(3, 4, 6, 7)**, the absorption of the neutral species at *λ*_max⁡_ = 402 − 427 nm vanished in 10^−1 ^M TFA, and new species with *λ*_max⁡  _ = 510 − 513 nm appeared. The absorption spectra of **4** in the presence of TFA show an isosbestic point at *λ* = 445 nm, as depicted in Figure 2. 

 As protonation of the pyrazine strongly enhances its electron-accepting capability, the D-A-D character of the chromophores becomes more pronounced, and the lowered LUMO results in shifts of the absorption maxima upto Δ*λ* = 86 − 107 nm (Δ*ν* = 5160 cm^−1^). The absorption maximum of the trimethoxy derivative **5** shifts only about Δ*λ* = 26 nm to the red, but an additional shoulder at *λ* = 490 nm results from protonation. Nevertheless, the basicity of the ground-state of these chromophores is nearly unbiased by the lateral donor groups. The bathochromic shifts of the absorption can be explained by an increased charge transfer from the donors to the pyrazinium moiety. This should be independent from the anion. 

**Figure 3:**
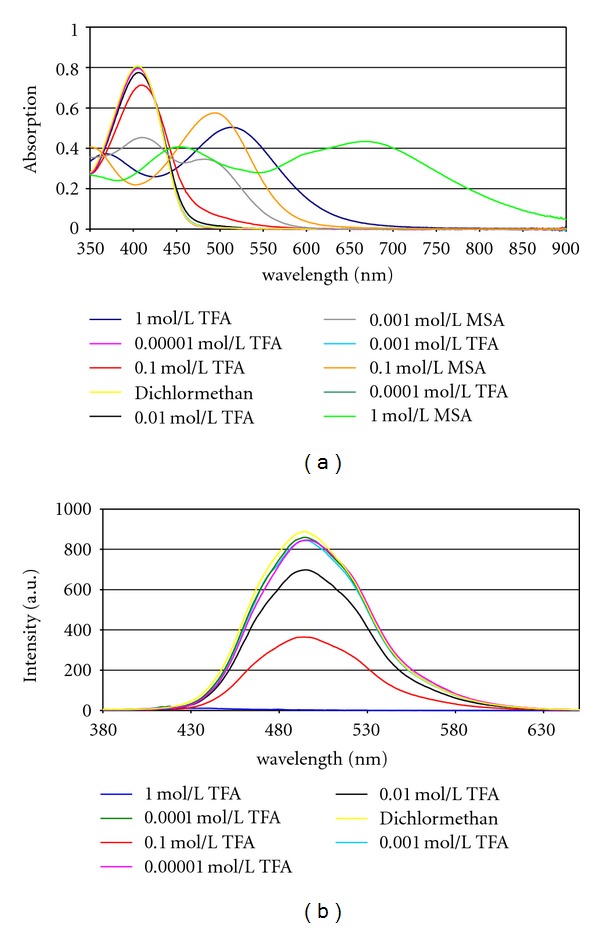
Acidochromism of absorption and fluorescence of bis(p-9-carbazolylstyryl)pyrazine **6** in the presence of TFA and MSA.

Using the stronger methanesulfonic acid (MSA) in DCM instead of TFA in DCM gives similar spectral results. With 10^−3^ M MSA nearly equimolar concentrations of neutral and protonated **6** are visible, and 0.1 M MSA results in only protonated **6**—but with *λ*_max⁡_ = 499 nm. A further increase in MSA concentration leads to a different absorption spectrum; the maximum at 499 nm vanishes and a broad band emanates in the red part, probably from a new, twofold protonated species. This band has a *λ*_max⁡_ = 665 nm and extends from the green into the NIR region with the absorption edge *λ*_0.1∗*λ*_max⁡__ > 900 nm. (Figure 3). The second protonation can occur at the second nitrogen atom of the pyrazine, but a protonation of the aromatic *π*-system cannot be excluded.

The angular distyrylpyrazines appear to have a higher basicity: protonation of **8**, **9** occurs in 10^−2^ M TFA. Both methoxystyryl units are in linear conjugation with one nitrogen atom of the pyrazine, thus increasing the basicity of this center. It should be noted that the V-shaped 3,4,5-trimethoxy derivative **10**, like the linear isomer **5**, behaves differently. 10^−2^ M TFA is sufficient to protonate **5**, **10**, but the displacements of the excitation bands are significantly smaller than those of the other chromophores except **2**. The latter compound carries two pyridine rings in the periphery; therefore, four basic sites appear on this molecule. Initial protonation occurs at the pyridine ring, probably converting a weak D-A-D electronic structure to a similarly weak A-D-A or A-A-D system, as the absorption spectrum is nearly unbiased by TFA. 

Brønsted acids have different effects on the emission of these fluorophores. Protonation of **1** results in a bathochromic shift of Δ*λ* = 79 nm (Δ*ν* = 3617 cm^−1^) starting at 10^−2^ M TFA and amounts to Δ*λ* = 103 nm (Δ*ν* = 4351 cm^−1^) in 1 M TFA. The acidochromic shift of the emission of the pyridine derivative **2** is much smaller, only Δ*λ* = 37 nm (Δ*ν* = 1901 cm^−1^) between neutral solution and 1 M TFA, but the protonation is already visible in 10^−3^ M TFA. Protonation of the linear methoxy-substituted compounds **(3–5)** results in gradually reduced emission and total quenching in the presence of 10^−1^ M TFA and higher (Figure 2), whereas the fluorescence of their angular counterparts **8–10** vanishes already in 10^−2^ M TFA. Like in their ground states, the excited states of the V-shaped dyes show higher basicities than the excited states of their linear isomers [37]. This is a result from the direct conjugation of *N*-1 of the pyrazine **(8 – 10)** with both donor-styryl segments and therefore enhanced charge transfer to this center. 

Among the donor-substituted dyes, **6** and **7** with carbazole end groups, are emissive even in the presence of high concentrations of TFA. The fluorescence of **6**, still moderate in 0.1 M TFA, is totally quenched only in 1 M TFA. **7**, the isomer of **6**, fluoresces with *λ*_max⁡_^F^ = 500 nm upto 10^−2^ M TFA. This emission is quenched by 0.1 M TFA, but a new emitting species appears when **7** is excited in 1 M TFA; a weak emission band with *λ*_max⁡_^F^ = 410 nm results from irradiation at 345 nm. Whereas phenylenevinylene chromophores are fairly stable towards light or acid, UV-irradiation of solutions with traces of acids initiates a fast and irreversible decomposition of these materials [38]. These styrylpyrazine chromophores show a much higher photostability, even in very acidic solutions.

## 4. Conclusion

The fluorescence of linear and angular distyrylpyrazines with a donor-acceptor-donor electronic structure is much more sensitive towards changes of the solvent polarity than their UV/Vis absorption spectra. The solvatochromic shifts of angular DSPs are higher than those of the linear isomers. Protonation with TFA results in strong bathochromic shifts of the absorption and reduced fluorescence intensity; in higher concentrations, the emission is quenched. But the carbazole-substituted DSPs are still fluorescent in 0.1 M TFA. Methanesulfonic acid gives a twofold protonated **6** with a broad absorption band extending into the NIR. These dyes can be used as colorimetric probes for acidity and as fluorescent sensors for polarity. Their quadrupolar electronic character **(1–7)**, solvatochromism of the emission, and extended *π*-system are good prerequisites for large two-photon absorption cross sections allowing a fluorescence sensing by NIR irradiation.

## 5. Experimental Part

*General Information*. All reactions were carried out under dry nitrogen unless otherwise indicated. Commercially available reagents were used without further purification unless otherwise indicated; solvents and gases were dried by standard procedures. Yields refer to chromatographically and sepctroscopically pure compounds unless otherwise stated. ^1^H and ^13^C NMR spectra were recorded with Bruker AC 300 (300 MHz), Bruker AV 400 (400 MHz), and Bruker ARX 400 (400 MHz) spectrometers in CDCl_3_, D_3_SO_4_, and DMSO-d_6_. The proton and carbon signals were assigned on the basis of DEPT, COSY 45, HMQC, and HMBC experiments. Chemical shifts are expressed as *δ* values in ppm; coupling constants are given in Hz. *Abbreviations*. Ph: phenyl, Cb: Carbazol, vin: vinylene, Pyr: pyrazine, sup.: superimposed signals. Melting points were determined by using a Büchi HWS SG 200. IR spectra were obtained on a JASCO 4100 FT-IR (ATR). FD-MS spectra were obtained on a Mat 95 (Finnigan); HR-ESI spectra were obtained on a Q-TOF-ULTIMA 3 with Lock Spray device (Waters-Micromass) and NaICsI Standard as reference. UV-vis spectra were recorded with Perkin-Elmer Lambda 16 and fluorescence spectra with Perkin-Elmer LS 50B. Elemental analyses were carried out by using a Vario EL. Semiempirical calculations of structures, dipole moments, and transitions were performed using the MOPAC package, optimization of structures with AM1, and electronic transitions INDO/S.

### 5.1. 4-(9-Carbazolyl) benzaldehyde 16

Carbazole (4.0 g, 23.9 mmol), 4-bromobenzaldehyde (4.5 g, 24 mmol), copper powder (0.3 g, 4.7 mmol), and potassium carbonate (3.3 g, 24 mmol) were suspended under nitrogen in o-dichlorobenzene (30 mL) and stirred for 3 d at 190°C. The cooled mixture was dissolved in ethyl acetate, filtered, and concentrated. The residue was recrystallized from toluene to yield 5.42 g (20 mmol, 84%) of a brownish crystalline solid with mp 162°C (Lit [39]: 163°C).

^1^H-NMR (CDCl_3_, 300 MHz): *δ* = 10.12 (s, 1H, H-1), 8.16 (d, ^3^ *J* = 8.6 Hz, 2H, H-10), 8.13 (d, ^3^ *J* = 8.3 Hz, 2H, H-3), 7.79 (d, ^3^ *J* = 8.3 Hz, 2H, H-4), 7.52 (d, ^3^ *J* = 7.9 Hz, 2H, H-7), 7.45 (t, 2H, H-8); 7.35 (t, 2H, H-9). IR (KBr): $\tilde{\nu }$/cm^−1^ = 3050, 2822, 2733, 1703, 1597, 1511, 1451, 1362, 1316, 1226, 1208, 1161, 914, 829, 751.

### 5.2. General Procedure for the Synthesis of Distyrylpyrazines

Dimethylpyrazine and the corresponding aldehyde were dissolved DMF in a 100 mL three-necked round bottom flask equipped with a stirrer and nitrogen inlet. The stirred mixture was cooled under nitrogen to 0°C and KO*t*Bu, added in small portions. After the addition of the base was complete, the mixture was brought to ambient temperature, and stirring was continued until all starting material had been consumed (TLC). Water was added, and the product was isolated by extraction with chloroform (3x). Washing of the chloroform solutions with brine (3x) and drying over Na_2_SO_4_ followed by evaporation of the solvent and recrystallization of the residue from chloroform/Methanol gave the pure products.

### 5.3. (E,E)-2,5-Distyrylpyrazine 1

According to the general procedure, 2,5-dimethylpyrazine **11** (180 mg, 1.66  mmol), benzaldehyde **12** (530 mg, 4.99 mmol), and potassium-*tert*-butanolate (560 mg, 4.99 mmol) in 30 mL DMF gave 260 mg (0.91 mmol, 55%) of a yellow plates with mp 225°C.

^1^H-NMR (CDCl_3_, 400 MHz): *δ* = 8.60 (s, 2H, 3-H, 6-H Pyr), 7.74 (d, ^3^ *J* = 16.1 Hz, 2H, 2-H vin), 7.60 (d, ^3^ *J* = 7.3 Hz, 4H, 2-H, 6-H Ph), 7.40 (t, 4H, 3-H, 5-H Ph), 7.33 (t, 2H, 4-H Ph), 7.18 (d, ^3^ *J *= 16.1 Hz, 2 H, 1-H vin). ^ 13^C-NMR (CDCl_3_, 100 MHz): *δ* = 149.1 (C-2, C-5 Pyr), 143.3 (C-3, C-6  Pyr), 136.2 (C-1 Ph), 134.4 (C-2 vin), 128.8 (C-3, C-4, C-5 Ph sup.), 127.3 (C-2, C-6 Ph), 124.0 (C-1 vin). FD-MS: m/z = 284.3 (100%) [M^+^]. IR (ATR): $\tilde{\nu }$/cm^−1^ = 1630, 1494, 1482, 1447, 1359, 1200, 1150, 1028, 971, 916, 754, 695. HR-ESI-MS: caldc for (C_20_H_16_N_2_ + H): 285.1392; found: 285.1404.

### 5.4. (*E*,*E*)-2,5-Bis[2-(3-pyridyl)ethenyl]pyrazine 2

Sodium (60 mg, 2.61 mmol) was dissolved in methanol (5 mL anhyd.) in a flame-dried flask under nitrogen, and dimethylpyrazine-di-*N*-oxide **19** (150 mg, 1.07 mmol) and pyridine carbaldehyde **18** (250 mg, 2.33 mmol) were added. After 24 h stirring at ambient temperature, the mixture was filtered, and the residue was recrystallized from acetic acid/methanol. Yield of di-N-oxide: 165 mg (49%), mp 200°C (dec.). ^1^H-NMR (D_2_SO_4_, 400 MHz): *δ* = 9.92 (s, 2H); 9.19 (s, 2H); 9.18 (d, 2H); 8.98 (d, 2H); 8.44 (t, 2H); 8.39 (d, 2H); 8.10 (d, 2H).

The di-N-oxide (60 mg, 0.19 mmol) was suspended in chloroform and cooled to 0°C. PCl_3_ (1 g, 7.28 mmol) was added dropwise, and the stirred mixture was refluxed for 5 h. Ice was added, and the mixture was neutralized with Na_2_CO_3_ and extracted with CHCl_3 _(5x). The organic solutions were washed with brine and dried (MgSO_4_), the solvent was evaporated, and the residue was recrystallized from dichloromethane/methanol to yield 35 mg (64%) of an off-white solid with mp 230°C (dec.).

^1^H-NMR (CDCl_3_, 400 MHz): *δ* = 8.80 (s, 2H, 3-H, 6-H Pyr), 8.60 (s, 2H, 2-H Py), 8.54 (d, ^3^ *J* = 4.4 Hz, 2H, 6-H Py), 7.90 (d, ^3^ *J* = 7.9 Hz, 2H, 4-H Py), 7.73 (d, ^3^ *J* = 16.1 Hz, 2H, 2-H vin), 7.32 (dd, ^3^ *J* = 4.4 Hz, ^3^ *J^'^*= 7.9 Hz, 2H, H-5-H Py), 7.23 (d, ^3^ *J* = 16.1 Hz, 2H, 1-H vin). ^13^C-NMR (CDCl_3_, 75 MHz): *δ* = 149.6, 149.1, 148.8 (C-3, C-6 Pyr, C-2, C-6 Py), 143.5 (C-2, C-2 Pyr), 133.5 (C-4 Py), 131.9 (C-3 Py), 131.0 (C-2 vin), 125.9 (C-1 vin), 123.7 (C-5 Py). FD-MS: m/z = 285.9 (100%) [M^+^]. IR (KBr): $\tilde{v}$/cm^−1^= 3027, 1631, 1569, 1492, 1417, 1360, 1153, 1030, 1023, 697, 803, 707, 636. IR (ATR): $\tilde{v}$/cm^−1^= 3062, 3020, 2988, 1625, 1517, 1397, 1354, 1217, 1190, 1142, 1012, 969, 937, 899, 792, 750, 698.

### 5.5. (E,E)-2,5-Bis(4′-methoxystyryl) pyrazine 3

According to the general procedure, 2,5-dimethylpyrazine **11** (108 mg, 1,0 mmol), 4-methoxybenzaldehyde **13** (272 mg, 2.0 mmol), and potassium-*tert*-butanolate (269 mg, 2.4 mmol) in 10 mL DMF gave 38 mg (0.11 mmol, 11%) of a yellow plates with mp 231°C.

^1^H-NMR (300 MHz, CDCl_3_): *δ* = 3.82 (s, 6 H, OCH_3_), 6.88 (d, ^3^ *J* = 8.7 Hz, 4 H, 3-H, 5-H Ph), 7.11 (d, ^3^ *J* = 15.6 Hz, 2 H, 1-H vin), 7.51 (d, ^3^ *J* = 8.7 Hz, 4 H, 2-H, 6-H Ph), 7.70 (d, ^3^ *J* = 15.6 Hz, 2 H, 2-H vin), 8.50 (s, 2 H, 3-H, 6-H Pyr). ^13^C-NMR (75.5 MHz, CDCl_3_): *δ* = 51.31 (OCH_3_), 111.22 (C-3, C-5 Ph), 118.97 (d), 124.70 (C-2, C-6 Ph), 127.81 (s), 131.99 (d), 139.07 (d), 149.76 (s), 152.69 (C-4 Ph). IR: (KBr): $\tilde{v}$/cm^−1^ = 3434, 2930, 2836, 1630, 1602, 1574, 1511, 1483, 1466, 1441, 1423, 1301, 1252, 1231, 1179, 1147, 1027, 982, 920, 859, 826, 752, 558, 513. FD-MS: m/z = 172.1 [M]^2+^ (1.4%), 344.2 [M]^+^ (100%). C_22_H_20_N_2_O_2_ calcd.: C 76.72, H 5.85, N 8.13; found C 76.46, H 5.65 N 8.30.

### 5.6. (E,E)-2,5-Bis(3′,4′-dimethoxystyryl) pyrazine 4

According to the general procedure, 2,5-dimethylpyrazine **11** (108 mg, 1,0 mmol), 3,4-dimethoxybenzaldehyde **14** (332 mg, 2.0 mmol), and potassium-*tert*-butanolate (269 mg, 2.4 mmol) in 10 mL DMF gave 200 mg (0.49 mmol, 49%) of a yellow plates with mp 225°C.

^1^H-NMR (300 MHz, CDCl_3_): *δ* = 3.87 (s, 6 H, 4-OCH_3_), 3.91 (s, 6 H, 3-OCH_3_), 6.84 (d, ^3^ *J* = 8.4 Hz, 2 H, 5-H Ph), 6.99 (d, ^3^ *J* = 15.9 Hz, 2 H, 1-H vin), 7.09–7.11 (m, 4H, 2-H, 6-H Ph), 7.61 (d, ^3^ *J* = 15.9 Hz, 2 H, 2-H vin), 8.51 (s, 2H, 3-H, 6-H Pyr). ^13^C-NMR (75.5 MHz, CDCl_3_): *δ* = 55.83 (3,4-OCH_3_), 109.01 (d), 111.14 (d), 121.13 (d), 122.07 (d), 129.27 (s), 133.71 (d), 142.85 (d), 148.81 (s), 149.06 (s), 149.79 (s). IR (KBr): $\tilde{v}$/cm^−1^ = 3432, 2931, 1627, 1598, 1514, 1466, 1441, 1421, 1324, 1259, 1158, 1141, 1023, 972, 816, 769, 612, 568. FD-MS: m/z = 404.1 [M]^+^ (100%). C_24_H_24_N_2_O_4_ calcd.: C 71.27, H 5.98, N 6.93; found C 70.88, H 5.95 N 6.90.

### 5.7. (E,E)-2,5-Bis(3′,4′,5′-trimethoxystyryl) pyrazine 5

According to the general procedure, 2,5-dimethylpyrazine **11** (108 mg, 1,0 mmol), 3,4,5-trimethoxybenzaldehyde **15** (432 mg, 5.1 mmol), and potassium-*tert*-butanolate (269 mg, 2.4 mmol) in 10 mL DMF gave 152 mg (0.33 mmol, 33%) of a yellow solid with mp 219°C

^1^H-NMR (300 MHz, CDCl_3_): *δ* = 3.87 (s, 6 H, 4-OCH_3_), 3.90 (s, 12 H, 3,5-OCH_3_), 6.81 (s, 4 H, 2-H, 6H Ph), 7.07 (d, ^3^ *J* = 16.2 Hz, 2 H, 1-H vin), 7.63 (d, ^3^ *J* = 16.2 Hz, 2 H, 2-H vin), 8.58 (s, 2 H, 3-H, 6-H Pyr). ^13^C-NMR (75.5 MHz, CDCl_3_): *δ* = 56.12, 60.97 (OCH_3_), 114.35 (C-2, C-6 Ph) 123.47 (d), 131.86 (s), 134.26 (d), 138.99 (d), 143.09 (s), 148.94 (s), 153.46 (s). IR (KBr): $\tilde{v}$/cm^−1^= 3433, 2933, 1631, 1582, 1464, 1450, 1420, 1322, 1275, 1238, 1128, 1004, 965, 804, 640. FD-MS: m/z = 232.1 [M]^2+^ (0.66%), 464.2 [M]^+^ (100%).

### 5.8. (E,E)-2,5-Bis[2-(4-(N-carbazolyl)phenyl) ethenyl] pyrazine 6

According to the general procedure, 2,5-dimethylpyrazine **11** (100 mg, 0.92 mmol), 4-(9-carbazolyl)benzaldehyde **16** (271 mg, 2.21 mmol), and potassium-*tert*-butanolate (250 mg, 2.23 mmol) in 50 mL DMF gave 20 mg (0.16 mmol, 4%) of a yellow solid with mp 266°C.

^1^H-NMR (CDCl_3_, 400 MHz): *δ* = 8.68 (s, 2H, 2-H, 6-H Pyr), 8.17 (d, ^3^ *J* = 7.7 Hz, 4H, 4-H, 5-H Cb), 7.88 (d, ^3^ *J* = 15.8 Hz, 2H, 2-H vin), 7.85 (d, ^3^ *J* = 8.4 Hz, 4 H, 2-H, 6-H, Ph), 7.64 (d, ^3^ *J* = 8.4 Hz, 4H, 3-H, 5-H Ph), 7.50 (d, ^3^ *J* = 7.5 Hz, 4H, 1-H, 8-H Cb), 7.45 (dt, 4H, 2-H, 7-H Cb), 7.32 (dt, 4H, 3-H, 5-H Cb), 7.29 (d, ^3^ *J* = 15.8 Hz, 2H, 1-H vin). ^13^C-NMR (CDCl_3_, 75 MHz): *δ* = 149.1 (C-2, C-5 Pyr), 143.6 (C-3, C-6 Pyr), 140.6 (C-1a, C-8a Cb), 138.1 (C-4 Ph), 135.2 (C-2 vin), 133.4 (C-2, C-6 Ph), 129.0 (C-1 Ph), 127.2 (C-1 vin), 126.1 (C-3, C-5 Ph), 124.7 (C-4 a,b Cb), 123.6 (C-2, C-7 Cb), 120.4, 120.2 (C-3, C-4, C-5, C-6 Cb), 109.8 (C-1, C-8 Cb). FD-MS: m/z = 307.2 (7.3%) [M^2+^], 613.9 (100%) [M^+^]. IR (ATR): $\tilde{v}$/cm^−1^ = 3055, 3030, 1597, 1511, 1477, 1449, 1358, 1334, 1315, 1228, 1148, 1027, 964, 816, 745, 722. HR-ESI-MS: calcd. for (C_44_H_30_N_4_ + H): 615.2549; found: 615.2558.

### 5.9. (E,E)-2,5-Bis[2-(3-(N-phenyl)carbazolyl)ethenyl]pyrazine 7

According to the general procedure, 2,5-dimethylpyrazine **11** (80 mg, 0.74 mmol), 9-phenylcarbazol-3-carbaldehyde **17** (600 mg, 2.21 mmol), and potassium-*tert.*-butanolate (250 mg, 2.23 mmol) in 50 mL DMF gave 85 mg (0.16 mmol, 19%) of a yellow solid with mp 250°C (dec.).

^1^H-NMR (CDCl_3_, 400 MHz): *δ* = 8.65 (s, 2H, 3-H, 6-H Pyr), 8.39 (s, 2H, 4-H Cb), 8.19 (d, ^3^ *J* = 7.7 Hz, 2H, 5-H Cb), 7.95 (d, ^3^ *J* = 16.1 Hz, 2H, 2-H vin), 7.70 (dd, ^3^ *J* = 8.6 Hz, ^4^ *J* = 1.5 Hz, 2H, 8-H Cb), 7.66–7.57 (m, 6H, 1-H Cb, 3-H, 5-H Ph), 7.49 (m, 2H, 7-H-Cb), 7.44-7.40 (m, 8H, 4-H Cb, 2-H, 4-H, 6-H Ph), 7.33 (m, 2H, 6-H Cb), 7.24 (d, ^3^ *J* = 16.1 Hz, 2 H, 1-H vin). ^13^C-NMR (CDCl_3_, 75 MHz): *δ* (ppm) = 149.4, 149.1 (C-2, C-3, C-4, C-5 Pyr), 143.0 (C-8a Cb), 141.4 (C-1a Cb), 137.4 (C-1 Ph), 134.8 (C-2 vin), 129.9 (C-12, C-6 Ph), 128.7 (C-3 Cb), 127.7 (C-4 Ph), 127.1 (C-3, C.5 Ph), 126.3 (C-7 Cb), 125.4 (C-2 Cb), 123.8 (C-4a Cb), 121.9 (C-1 Vin, C-4b Cb), 120.4, 119.6 (C-4, C-5, C-6 Cb), 110.2, 110.0 (C-8 Cb, C-2, C-.6 Ph). FD-MS: m/z = 614.2 (100%) [M^+^]. IR (KBr): $\tilde{v}$/cm^−1^ = 3045, 3012, 2968, 1621, 1596, 1452, 1359, 1234, 1188, 1147, 1026, 966, 802, 744, 698. HR-ESI-MS calcd. for (C_44_H_30_N_4_ + H): 615.2549, found; 615.2560.

### 5.10. (E,E)-2,6-Bis(4′-methoxystyryl)pyrazine 8

According to the general procedure, 2,6-dimethylpyrazine **20** (108 mg, 1.0 mmol), 4-methoxybenzaldehyde **13** (340 mg, 2.5 mmol), and potassium-*tert*-butanolate (390 mg, 3.48 mmol) in 15 mL DMF gave 115 mg (0.34 mmol, 34%) of a yellow solid with mp 169°C.

^1^H-NMR (300 MHz, CDCl_3_): *δ* = 3.83 (s, 6 H, OCH_3_), 6.92 (d, ^3^ *J* = 8.7 Hz, 4 H, 3-H, 5-H Ph), 7.02 (d, ^3^ *J* = 15.9 Hz, 2 H, 1-H vin), 7.55 (d, ^3^ *J* = 8.7 Hz, 4 H, 2-H, 6-H Ph), 7.75 (d, ^3^ *J* = 15.9, 2 H, 2-H vin), 8.39 (s, 2 H, 3-H, 5-H Pyr). ^13^C-NMR (75.5 MHz, CDCl_3_): *δ* = 55.33 (OCH_3_), 114.25 (C-3, C-5 Ph), 122.29 (d), 128.72 (C-2, C-6 Ph), 129.05 (s) 134.49 (d), 140.97 (d), 150.58 (s), 160.24 (C-4 Ph). IR: (KBr): $\tilde{v}$/cm^−1^ = 3434, 2928, 2832, 1630, 1601, 1579, 1511, 1469, 1441, 1418, 1311, 1254, 1175, 1147, 1031, 972, 859, 752, 558, 513. FD-MS: m/z = 172.1 [M]^2+^ (0.6%), 344.2 [M]^+^ (100%). C_22_H_20_N_2_O_2_ calcd.: C 76.72, H 5.85, N 8.13; found C 76.31, H 5.84 N 7.76.

### 5.11. (E,E)-2,6-Bis(3^'^,4^'^-dimethoxystyryl)pyrazine 9

According to the general procedure, 2,6-dimethylpyrazine **20** (108 mg, 1.0 mmol), 3,4-dimethoxybenzaldehyde **14** (310 mg, 2.2 mmol), and potassium-*tert.*-butanolate (310 mg, 2.8 mmol) in 15 mL DMF gave 155 mg (0.41 mmol, 41%) of a yellow solid with mp 208°C.

^1^H-NMR (300 MHz, CDCl_3_): *δ* = 3.83 (s, 6 H, 4-OCH_3_), 3.90 (s, 6 H, 3-OCH_3_), 6.87 (d, ^3^ *J* = 8.4 Hz, 2 H, 5-H Ph), 6.99 (d, ^3^ *J* = 15.9 Hz, 2 H, 1-H vin), 7.10–7.12 (m, 4 H, 2-H, 6-H Ph), 7.71 (d, ^3^ *J* = 15.9 Hz, 2 H, 2-H vin), 8.41 (s, 2 H, 3-H, 5-H Pyr). ^13^C-NMR (75.5 MHz, CDCl_3_): *δ* = 55.90, 55.95 (3,4-OCH_3_), 109.11, 111.13 (C-2, C-5 Ph), 121.27 (d), 122.18 (d) 129.38 (s), 133.90 (d), 134.77 (d), 142.95 (s), 149.19, 149.22 (s). IR (KBr): $\tilde{v}$/cm^−1^ = 3446, 2927, 2830, 1636, 1599, 1508, 1457, 1419, 1265, 1218, 1164, 1140, 1031, 1020, 974, 854, 799, 754, 552. FD-MS: m/z = 404.3 [M]^+^. C_24_H_24_N_2_O_4_ calcd.: C 71.27, H 5.98, N 6.93; found C 70.87, H 6.54 N 6.81.

### 5.12. (E,E)-2,6-Bis(3′,4′,5′-trimethoxystyryl)pyrazine 10

According to the general procedure, 2,6-dimethylpyrazine **20** (108 mg, 1.0 mmol), 3,4,5-trimethoxybenzaldehyde **15** (490 mg, 2.5 mmol), and potassium-*tert.*-butanolate (393 mg, 3.5 mmol) in 15 mL DMF gave 177 mg (0.39 mmol, 39%) of a yellow solid with mp 206°C. ^1^H-NMR (300 MHz, CDCl_3_): *δ* = 3.88 (s, 6 H, *p*-OCH_3_), 3.92 (s, 12 H, 3,5-OCH_3_), 6.85 (s, 4 H, 2-H, 6-H Ph), 7.08 (d, ^3^ *J* = 15.9 Hz, 2 H, 1-H vin), 7.71 (d, ^3^ *J* = 15.9 Hz, 2 H, 2-H vin), 8.48 (s, 2 H, 3-H, 5-H Pyr). ^13^C-NMR (75.5 MHz, CDCl_3_): *δ* = 56.29 (3,4,5-OCH_3_), 116.36 (C-2, C-6 Ph), 123.59 (d), 132.64 (s), 134.29 (d), 139.65 (d), 144.23 (s), 149.84, 151.31 (s). IR (KBr): $\tilde{v}$/cm^−1^ = 3447, 2940, 2831, 1636, 1581, 1506, 1421, 1338, 1320, 1274, 1238, 1127, 1005, 955, 854, 811, 594, 464. FD-MS: m/z = 464.4 [M]^+^ (100%), 928.8 [M_2_]^+^ (1%). C_26_H_28_N_2_O_6_ calcd.: C 67.23, H 6.08, N 6.03; found H 6.06, N 6.08.

## Figures and Tables

**Scheme 1:**
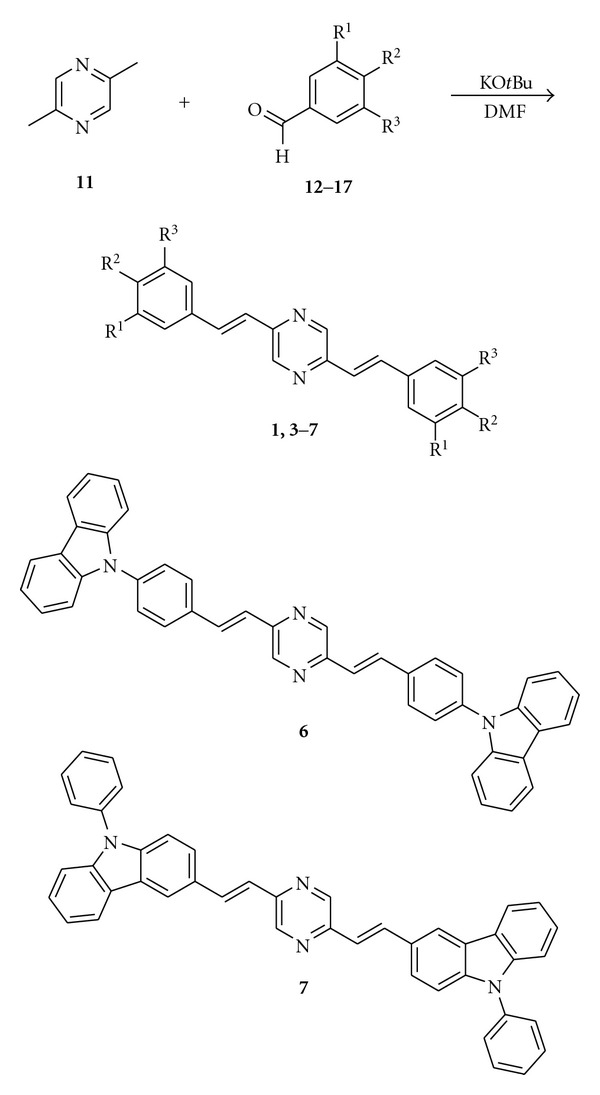
Synthesis of linear distyrylpyrazines and structures of DSPs with carbazole end groups.

**Table 1:** Substitution pattern of linear and angular distyrylpyrazines.

Entry	R^1^	R^2^	R^3^	Yield	Color	m.p.
**1**	H	H	H	55%	yellow	225°C
**2**	(*β*-pyridyl)	H	H	64%	whitish	230°C
**3**	H	OCH_3_	H	11%	yellow	231°C
**4**	OCH_3_	OCH_3_	H	49%	yellow	225°C
**5**	OCH_3_	OCH_3_	OCH_3_	33%,	orange	219°C
**6**	H	9-Carbazolyl	H	4%	yellow	266°C
**7**	N-Phenylindolo	N-Phenylindolo	H	19%	yellow	250°C dec.
**8**	H	OCH_3_	H	34 %	yellow	169°C
**9**	OCH_3_	OCH_3_	H	41%	yellow	208°C
**10**	OCH_3_	OCH_3_	OCH_3_	39%	yellow	206°C

**Table 2:** Solvatochromism of distyrylpyrazines **1–10**, *λ*_*max*_/nm, *ε* /L mol^−1^cm^−1^; *E*_*I*_^*N*^ [35].

Comp.		Cyclohexane	Toluene	Dichloromethane	Acetonitrile	Ethanol	*ε*_max⁡_ (DCM)
	E_I_^N^	0.006	0.099	0.309	0.460	0.654	
**1 **	*λ*_max⁡_	382	386	383	379	381	43537
	*λ*_max⁡_^F^	424	428	432	429	443	
**2 **	*λ*_max⁡_	376	379	377	373	374	46865
	*λ*_max⁡_^F^	415	422	423	419	428	
**3 **	*λ*_max⁡_	399	405	402	397	400	
	*λ*_max⁡_^F^	439	453	465	463	482	
**4 **	*λ*_max⁡_	407	413	409	407	407	
	*λ*_max⁡_^F^	448	463	476	478	501	
**5 **	*λ*_max⁡_	402	406	402	399	400	
	*λ*_max⁡_^F^	449	463	489	499	514	
**6 **	*λ*_max⁡_	404	408	405	398	403	61976
	*λ*_max⁡_^F^	451	463	495	504	520	
**7 **	*λ*_max⁡_	423	427	427	420	423	58692
	*λ*_max⁡_^F^	464	478	501	501	517	
**8 **	*λ*_max⁡_	400	404	400	397	400	
	*λ*_max⁡_^F^	440	453	463	462	482	
**9 **	*λ*_max⁡_	391	397	395	395	397	
	*λ*_max⁡_^F^	427	443	461	471	498	
**10 **	*λ*_max⁡_	376	380	381	383	382	
	*λ*_max⁡_^F^	424	446	474	495	513	

**Table 3:** Acidochromism of distyrylpyrazines **1–10** in dichloromethane and dichloromethane/TFA.

Entry	*λ*_max⁡_ DCM	*λ*_max⁡_ 10^−5^ M TFA	*λ*_max⁡_ 10^−4^ M TFA	*λ*_max⁡_ 10^−3^ M TFA	*λ*_max⁡_ 10^−2^ M TFA	*λ*_max⁡_ 10^−1^ M TFA	*λ*_max⁡_ 1 M TFA
**1** *λ*_max⁡_	383	383	383	386	400	433	450
*λ*_max⁡_^F^	432	432	432	433	512	524	535
**2** *λ*_max⁡_	377	376	377	374	373	371	379
*λ*_max⁡_^F^	423	423	423	439	445	445	460
**3** *λ*_max⁡_	402	399	403	402	402	484	501
*λ*_max⁡_^F^	465	464	465	465	463		
**4** *λ*_max⁡_	409	409	410	410	411	498	506
*λ*_max⁡_^F^	476	477	477	478	477		
**5** *λ*_max⁡_	402	401	402	403	424	392 (490 sh)	428 (490 sh)
*λ*_max⁡_^F^	489	491	488	490	488		
**6** *λ*_max⁡_	405	405	405	405	408	408	512
*λ*_max⁡_^F^	495	495	495	495	495	495	
**7** *λ*_max⁡_	427	427	427	427	432	532	513
*λ*_max⁡_^F^	501	501	501	501	500		410
**8** *λ*_max⁡_	400	399	400	407	468	489	499
*λ*_max⁡_^F^	463	463	462	462			
**9** *λ*_max⁡_	395	396	398	407	476	489	502
*λ*_max⁡_^F^	461	463	463	462			
**10** *λ*_max⁡_	381	378	381	386	401	444	451
*λ*_max⁡_^F^	474	476	476	477			
